# Developing Organizational Diversity Statements Through Dialogical Clinical Ethics Support: The Role of the Clinical Ethicist

**DOI:** 10.1007/s11673-023-10258-3

**Published:** 2023-05-26

**Authors:** Charlotte Kröger, Albert C. Molewijk, Suzanne Metselaar

**Affiliations:** 1 Department of Ethics, Law and Humanities, Amsterdam Public Health research institute, Amsterdam UMC, Vrije Universiteit Amsterdam, De Boelelaan 1089a, 1081 HV Amsterdam, Netherlands; 2 Centre for Medical Ethics, University of Oslo, Oslo, Norway; 3 Faculty of Military Sciences, Netherlands Defence Academy, Breda, The Netherlands

**Keywords:** Diversity statements, Clinical ethics support, Socratic dialogue, Participation, Inclusion, Healthcare organizations, Social justice

## Abstract

In pluralist societies, stakeholders in healthcare may have different experiences of and moral perspectives on health, well-being, and good care. Increasing cultural, religious, sexual, and gender diversity among both patients and healthcare professionals requires healthcare organizations to address these differences. Addressing diversity, however, comes with inherent moral challenges; for example, regarding how to deal with healthcare disparities between minoritized and majoritized patients or how to accommodate different healthcare needs and values. Diversity statements are an important strategy for healthcare organizations to define their normative ideas with respect to diversity and to establish a point of departure for concrete diversity approaches. We argue that healthcare organizations ought to develop diversity statements in a participatory and inclusive way in order to promote social justice. Furthermore, we maintain that clinical ethicists can support healthcare organizations in developing diversity statements in a more participatory way by fostering reflective dialogues through clinical ethics support. We will use a case example from our own practice to explore what such a developmental process may look like. We will critically reflect on the procedural strengths and challenges as well as on the role of the clinical ethicist in this example.

In increasingly pluralist societies, healthcare organizations struggle to find ways to foster a good, equitable, and inclusive care and work environment for all (Carnes and Sheridan 2019; Hooge et al. 2009; Waisel 2013). Structural inequalities exist in access, use, and provision of healthcare services (Barrett and Wholihan 2016; Chance, 2013; Johnstone and Kanitsaki 2009; Cohen, Gabriel, and Terell 2002). Also, quality of care and care outcomes are lower for minoritized patients (Bakhtiari, Olaffsdottir, and Beckfield 2018; Seeleman, Essink-Bot, Stronks, and Ingleby 2015)—we use the term “minoritized” here in recognition of minority status being a social construct. Further, there is unequal gender and minority representation in the workforce, especially in higher management positions (Cohen, Gabriel, and Terell 2002; Frusti, Niesen, and Campion 2003; Gathers 2003; LaVeist and Pierre 2014; Phillips and Malone 2014; Williams et al. 2014). Additionally, organizational constraints, like scarcity of time and resources, can negatively affect the way healthcare organizations acknowledge, address, and accommodate diversity in both practice and policy (Celik et al. 2008).

Diversity is a multifaceted concept that can be defined in various ways (Ewijk, 2011; Kröger et al. 2022). A prerequisite for thinking about equity in healthcare organizations is the recognition that social identities are constructed of a variety of social categories, including parameters like race, gender, ability, and class, that overlap and interact to create heterogeneous loci of marginalization, discrimination, and disadvantage (Cattacin, Chiarenza, and Domenig 2013). In this paper, we focus on differences in cultural and religious backgrounds, gender identity, and sexual orientation which may interact with each other and with other categories of difference. We chose these parameters specifically because they have been linked to a variety of care disparities (Bakhtiari, Olaffsdottir, and Beckfield 2018; Barrett and Wholihan 2016; Johnstone and Kanitsaki 2009), which makes them important dimensions within the broader concept of diversity in healthcare settings. Additionally, the healthcare organization we describe in our case example at a later stage identified these aspects of diversity as being linked to recurring and specific moral challenges related to equity and social justice in daily healthcare practice.

Diversity in the healthcare workforce and patient populations also means that different value-systems and moral perspectives on health, well-being, or good care exist (Blumberga and Safonova 2016; Inguaggiato, Metselaar, Porz, and Widdershoven 2019; Warnes et al. 2004). Hence, we need to consider the role of individuals’ social locations and systemic inequalities associated with cultural and religious backgrounds, gender identity, and sexual orientation, among other things, to make care environments and institutions more just. Healthcare organizations increasingly face the challenge to address diversity in order to facilitate equitable care and work practices in the diversifying healthcare landscape (see also Charlesworth 2005; Chattopadhyay and De Vries 2013; Cohen, Gabriel, and Terrell 2002). Organizational “diversity-responsiveness” refers to practices that foster and support diversity in the workforce and enable care access and good quality of care for *all* patients.

Developing a diversity statement or policy is such a practice; it can be an important starting point from which to promote diversity-responsive strategies, awareness, and equitable care practices within healthcare organizations (see for instance Carnes and Sheridan 2019; Leyerzapf et al. 2019; Phillips and Malone 2014; Seeleman et al. 2014, 2015; Yearwood et al. 2006). Diversity statements describe normative ideas and moral values regarding how diversity is defined and act as a formal “moral compass” or declaration of intent concerning an organizations’ diversity approach. Ideally, this also includes insight on how the organization will actually approach diversity-related moral challenges in practice, like inequalities in care access or minority representation in leadership positions (Denier and Gastmans, 2013; Gündemir et al. 2017).

The focus on normative assumptions and dimensions of organizational life, diversity, and good care practices makes the development of a diversity statement or policy as such an inherently ethical process, i.e., a process of moral discourse and reflection in which different values about good practices related to diversity are shared and explicated (see also Gallagher and Goodstein 2002 on organizational ethics and mission discernment in healthcare organizations). As clinical ethicists are engaged with ethical issues and ethical deliberation in healthcare organizations, it is therefore a process in which the ethicist may provide support. Here, we argue that clinical ethicists can support healthcare organizations with developing diversity statements in a participatory way through CES that fosters joint, dialogical reflection.

Clinical ethicists engage in different forms of clinical ethics support (CES) to support healthcare professionals and organizations with a variety of moral questions and dilemmas. Certain approaches to CES specifically strive to facilitate deliberative dialogical practices among various stakeholders in which different norms and values can be explored openly in order to foster moral learning and improve (care) practices (Abma et al. 2009; Metselaar et al. 2017; Molewijk, Slowther, and Aulisio 2016; Pratt and DeVries 2018). However, an exploration of how exactly clinical ethicists can employ dialogical CES methods to support healthcare organizations in developing diversity statements in a participatory way is still lacking in academic literature.

In this article, we provide such an exploration. First, we will address several moral challenges related to formulating diversity statements in healthcare organizations. Then we will describe how perspectives on participation can inform dialogical approaches to CES. Subsequently we will provide a case example in which we, as ethicists, employed such a dialogical approach to CES through engaging in a dialogical process that consisted of a Socratic dialogue, two Socratic explorations, and external stakeholder consultation, to facilitate the development of a diversity statement for a long-term care organization in a metropolitan area in the Netherlands. In the discussion, we critically analyse this development process. Furthermore, we will reflect on the role of the clinical ethicist when taking a participatory approach to CES to support the development of diversity statements in healthcare organizations.

## Developing Diversity Statements

There is growing acknowledgement in organizations of the importance of diversity statements and policies to promote diversity-responsive and inclusive practices and strategies. This is for instance seen in the Diversity Charter Platform of the European Commission: a network of the diversity charters of twenty-six European countries. Each charter acts as a declaration of intent for organizations to promote, develop, and implement diversity and inclusion strategies. In the Netherlands, 342 organizations have currently signed the Dutch Diversity Charter (Diversiteit in Bedrijf n.d.). Diversity statements and policies can concern various aspects of diversity. In healthcare organizations they may focus on workforce recruitment and on increasing the access to and quality of care for minoritized patients (Carnes and Sheridan 2019; Leyerzapf 2019). Diversity statements can provide moral reasons (like social justice) or business-case reasons (like productivity) for promoting diversity and inclusion, or a combination of both (Jansen et al. 2021). In this section we argue for engaging in a participatory process when developing diversity statements in healthcare organizations.

Developing diversity statements in healthcare organizations is a challenging endeavour. First, formulating the *content* of a normative statement about diversity is in itself complex, especially within pluralist societies. A challenge lies in ensuring that such a statement is inclusive, respects cultural and moral diversity and traditions, and can be realized in practice without undesired effects (Carnes and Sheridan 2019). For example, certain arguments for diversity may make an organization less attractive to existing or to prospective employees, also to those that it desires to reach in order to enhance equity and diversity in the workforce (Carnes and Sheridan 2019; Jansen et al. 2021). Further, if diversity statements focus on specific target groups only rather than taking an intersectional approach, this prioritization may lead to inequalities or even stereotyping (see Cattacin, Chiarenza, and Domenig 2013 who address this in the context of equity standards).

Second, there is the issue regarding what a good *process* for developing a diversity statement in healthcare organizations looks like. This issue will be the focus of this article. Despite the organizational trend towards *having* diversity statements, Ghorashi and Sabelis (2013) have maintained that many organizations—also outside the healthcare context—continue to create and reinforce old habits “of exclusion and isolation along the lines of ethnicity, gender, class age and other dimensions” through policy, management, and in practice, usually as a result of “power dynamics” (80). The privilege of dominant groups makes it almost impossible in organizations for women and minoritized individuals to be included and to contribute equally in organizational decision-making processes, including the development of diversity statements (see also Gathers 2003).

Traditionally, when developing policies such as diversity statements, those that already possess positions of power in healthcare tend to make decisions about those that do not (Denier and Gastmans 2013; Denier et al. 2019). However, other authors argue that for reasons pertaining to equality and social justice, the process of developing diversity statements, policies, or practices in organizations ought to be based on inclusive grounds, by “embracing all talent and reaching out to diverse groups that traditionally were not part of the core of organizations” (Ghorashi and Sabelis 2013, 78).

Additionally, other scholars have maintained that the participation of a diverse group of stakeholders in the process of formulating and implementing diversity-responsive care, strategies, and policies in healthcare is significant in fostering equity and inclusion (Abma et al. 2017a, b; Frusti, Niesen, and Campion 2003; Leyerzapf et al. 2019). Seeleman et al. (2015) have contended that healthcare organizations ought to enable patients and communities to participate in the “planning, developing and delivering” of healthcare services, policies, and strategies, given as these concern the patients and communities themselves (15). Frusti, Niesen, and Campion (2003) have argued that fostering diversity competence in organizations is a long-term commitment and that a diverse group of the workforce ought to be involved in the development of diversity initiatives. Furthermore, Ghorashi and Sabelis (2013) state that an important prerequisite for approaching diversity in organizations is to create a discursive space in which “others” are included instead of marginalized and power relationships are unravelled and where individuals can share insight—“listen to each other and grow closer” (83).

Healthcare organizations ought to engage in a participatory process when establishing diversity statements in order to address concerns of exclusion and power differences in policy and practice. To facilitate diversity-responsiveness, all relevant voices ought to be included, not only the dominant ones (Abma et al. 2017a, b; Seeleman et al. 2015). Therefore, rather than formulating diversity statements or policies only with those that already hold positions of power, like policymakers or management, those that are specifically affected by the policy should be involved as well (Ghorashi and Sabelis 2013). In healthcare these stakeholders may pertain to various healthcare professionals, patients, and communities.

Participatory practice does not (intend to) take away all moral challenges that exist in healthcare organizations when developing or implementing diversity statements. Rather, it may bring diverse perspectives and value conflicts to light—about different interpretations and experiences of good care or diversity practices, for instance. Further, stakeholder participation may help to address moral normativity or values that may otherwise (unknowingly) be imposed from one group (e.g., majority) onto the other (e.g., minority). Participatory practices may also contribute to combatting window-dressing, i.e., simply *having* an organizational diversity statement as an end in itself rather than with the intention to identify and achieve necessary social change. We maintain that the clinical ethicist may play an important role in designing and facilitating such a participatory process to establish a diversity statement in a healthcare organization.

## Moral Case Deliberation: A Participatory Approach to Clinical Ethics Support

Clinical ethicists provide clinical ethics support (CES) to facilitate healthcare professionals in dealing with their moral issues in healthcare practice (Molewijk et al. 2016). While various forms of CES exist, some types are based on dialogical and participatory principles—that is, equality, open discourse, and the inclusion of all voices—such as moral case deliberation (MCD). MCD encompasses different, methodically structured methods of CES that foster joint, dialogical reflection among a—preferably multidisciplinary—group of healthcare professionals on a real case that is experienced as morally troublesome (Molewijk et al. 2008; Molewijk et al. 2016).

The philosophical principles of MCD are based on pragmatic, hermeneutic, and dialogical ethics (Widdershoven and Molewijk 2010). MCD proceeds from the idea that in CES, moral questions and dilemmas ought to be explored dialogically, through a process of joint moral deliberation among a group of participants in which their moral intuitions and judgements are explicated on the basis of a practical case (Abma et al. 2009; Abma, Leyerzapf & Landeweer 2016; Dauwerse et al. 2011; Rasoal et al. 2017). A core aspect of MCD is that what is morally right cannot be decided on an abstract level. Instead, the *lived experience* of healthcare professionals is regarded to be the main source of moral knowledge and the decisive point of reference (Abma et al. 2009). This approach has similarities to literature on participatory health research, where knowledge is not seen as fixed but exists in dialectic interaction that incorporates multiple perspectives and types of knowing (Springett, Wright, and Roche 2011).

An MCD is facilitated either by an ethicist or a trained healthcare professional (Plantinga et al. 2012; Stolper et al. 2015). The facilitator helps the participants to focus on their concrete moral experiences, while structuring the deliberation process and looking after the optimal conditions for the dialogue, which include equality among participants, the postponement of prejudices, and active listening. By employing MCD, she helps participants with explicating their implicit values, norms, and perspectives (Widdershoven and Metselaar 2012). This allows healthcare professionals to make a morally well-founded choice for a course of action, together.

Furthermore, MCD seeks to improve the moral learning and moral competencies of participants and can be employed to reflect on the moral quality of care at an institutional or organizational level. In a MCD session, including a multitude of perspectives (from healthcare professionals, managers, patients, and their relatives, etc.) is considered beneficial to the moral quality of the deliberation, and the expertise of the participants and the ethicist is seen as complementary.

One type of MCD that clinical ethicists may employ to foster participatory, dialectic reflection in healthcare is the Socratic dialogue (SD). The SD is a structured method of deliberating about a practical philosophical question on the basis of concrete experiences within a group (Steinkamp and Gordijn 2003, 243). It is a form of MCD that is directed at gaining experience-based philosophical insight. Based on Socrates and later redeveloped by Nelson for educational settings (1922), contemporary versions of SD start with a philosophically relevant question about “the good.” This question is then reflected upon by referring to concrete, personal experiences of various participants whilst focusing on one specific case in particular. Then general rules and principles are abstracted from this case in order to, finally, define an answer to the starting question.

A key component of SD is the Socratic method, which entails asking participants to describe, explore, and defend their moral intuitions in order to reveal (pre-)judgements and character and to gain moral insight through collective, interactive, and critical deliberation (Birnbacher 1999). The SD is founded on the fundamental epistemological assumption that to gain moral insight, one ought to recognize one’s own lack of knowledge and ignorance and ask “sincere questions as the core of dialogue” (Stolper, Molewijk, and Widdershoven 2015, 49). By engaging in a dialogue, venturing into others’ perspectives, and asking open, earnest, and curious questions in a group setting, it is possible to discover more general moral insights about a moral question, also within organizations (Kessels 1994, 1997; Skordoulis and Dawson 2007; Widdershoven 2001). Thus, the emphasis lies on gaining moral insight and learning through democratic, collaborative interaction.

A central assumption underlying the SD is that “every human being has equal access to truth, provided every participant in a dialogue is committed to a disciplined group deliberation” (Steinkamp and Gordijn 2003, 243). This means that moral insight is not reserved to those trained in philosophy or those considered authorities but to everyone that participates in a democratic deliberation. In a SD, truth is understood as context-dependent and based on the experiences of the individuals participating in the dialogue.

This makes the SD a useful method to tackle epistemic injustice: the situation when someone’s knowledge is not taken seriously or into account due to their (perceived) group membership (Fricker 2007), in a dialogical context. A SD can minimize hierarchy and power relationships through dialogue. The central tenets of equality, lack of authority in truth-finding, democratic deliberation, not knowing, and personal experiences makes the SD a method that can be used to foster inclusive and participatory dialogical reflection.

The clinical ethicist has a key role in stimulating equal participation when facilitating SD as a form of CES. She guides the reflection process, enforces procedural rules, facilitates consensus, and ensures a diversified, open, and consensus-oriented thought process to promote participation and mutual understanding (Birnbacher 1999; Kessels 1997).

To safeguard an equal, power-free, and inclusive process neither the facilitator nor the participants should invoke personal authority and try to influence or dominate other participants. This is necessary to enable participants to feel safe, to be open to each other, and to earnestly question their own and others’ perspectives (Birnbacher 1999). It ensures that all perspectives are taken into account equally so that consensus is attained through a joint effort that involves active participation of all. This makes SD a “practical tool to facilitate “participative” change” (Skordoulis and Dawson 2007, 991) and a useful method for reflection on diversity issues in (healthcare) organizations. However, using the SD in CES in order to support healthcare organizations in developing diversity statements in a participatory way has not yet been explored.

## Case Example: Facilitating SD and Socratic Explorations to Develop a Diversity Statement in a Participatory Way

In this section we will discuss a case example from our own practice as ethicists. We will illustrate how clinical ethicists, by way of a Socratic approach, may support healthcare organizations in developing a diversity statement in a more participatory way. By a Socratic approach, we refer to a process in which the clinical ethicists facilitated dialogical and experience-based reflection (Socratic explorations) among a group of participants and engaged in additional activities to include further stakeholders. In this process, the Socratic method and having a Socratic attitude of not-knowing and asking sincere questions, rather than providing mere opinions, and inquiring about each other’s judgements and perspectives dialogically and through curious questions was the key deliberative technique. In the subsequent case example, we will not analyse the content of the diversity statement as such but we will describe the explorative, dialogical process, and participatory influences that led to the formulation of the statement. Our aim is to illustrate a first attempt of how dialogical CES can be employed to strive for more participation and inclusion when developing organizational diversity statements. We have notified the director we collaborated with about this publication.

### Context and Background

In 2017, the director of the elderly care division of a large long-term care organization in the Netherlands asked the first and last author as clinical ethicists to facilitate dialogical reflection on the question “what is a good way of dealing with diversity in elderly care?” The clinical ethicists had and still have a long-standing relationship with this organization. They regularly provide CES through trainings, workshops, and reflections on different moral questions in care and leadership to various healthcare professionals and managers. The aim was to reach consensus on good diversity practices in elderly care and to develop a diversity statement for the elderly care sector in a participatory and dialogical way. This statement was later adopted by the board of directors as a guideline for an official diversity policy for the whole organization, pertaining to care practices and to recruiting and supporting employees. The healthcare organization had identified cultural, religious, and sexual diversity as central aspects of diversity to be addressed in their recruitment strategies and care practices in order to tackle social inequities that they were confronted with a great deal in daily practice. Therefore, the clinical ethicists were asked to focus on these aspects of diversity in particular during their dialogical quest for developing a diversity statement together with stakeholders.

**Figure 1:**
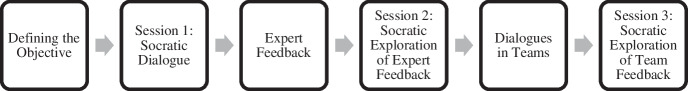
Overview of the Socratic Process to Develop the Diversity Statement

As clinical ethicists, we engaged in a dialogical process that consisted of three Socratic sessions and additional stakeholder consultation. During the first session we facilitated a SD (Kessels 1997). The second and third session were structured as Socratic explorations in which we facilitated dialogical reflection through the Socratic method and continuously stressed a Socratic attitude among all participants. This allowed all participants to engage in a safe and inclusive dialogue in which they were enabled to show openness and willingness to venture into each other’s perspectives and to question and reflect on their own judgements. Between the three sessions additional stakeholders participated in dialogical reflections and provided feedback. The Socratic process took place over six months and is visualized in figure 1.

All participants, mostly team managers, worked at different elderly care locations within the organization. They were recruited by the director (who was also the diversity officer of the organization) based on their interest in and affiliation with diversity and inclusion in care. The strategic position of team managers within the organization, standing between patients, other healthcare professionals, and higher management or directors, made them a good core group for the SD and Socratic explorations. In between the three dialogical sessions, they engaged in dialogues with their teams on temporary outcomes of these sessions facilitated by the ethicists. Additionally, researchers with relevant expertise in diversity issues were consulted to provide feedback on preliminary results of the process in order to refine the outcome of the first session. The process is summarized in table 1.

**Table 1:** Process Description of the Socratic Process to Develop a Diversity Statement

Activity	Description	Stakeholders	Facilitator(s)	Main outcome
Defining the Objective	Conversation with director about the objective, approach, and time frame	Director	CK and SM	Diversity is defined as related to sexuality, culture, and religion
Session 1: Socratic Dialogue	Socratic dialogue on “what is a good way of dealing with diversity in the elderly care sector?”	Twelve team managers and their director	CK and SM	Preliminary answer to starting question: definition
Expert Feedback	Experts provided written feedback on the outcome of Session 1	Five experienced researchers	None	Criticism: strengths and weaknesses of preliminary definition (1)
Session 2: Socratic Exploration of Expert Feedback	Reflection on expert feedback through Socratic method	Twelve team managers and their director, two patient representatives, one policy advisor	CK and SM	Adoption and discarding of elements of expert feedback. Preliminary definition (2)
Dialogues in Teams	Dialogues within eight teams on outcome Session 2	Care team members of eight team managers	Eight Team managers	Criticism: strengths and weaknesses of preliminary definition (2)
Session 3: Socratic Exploration of Team Feedback	Reflection on team feedback through Socratic method	Twelve team managers and their director, two patient representatives, one policy advisor	CK and SM	Adoption and discarding of elements of team feedback. Final definition.

### The Socratic Process

Before engaging with the actual Socratic process itself, the first and last author met with the director of the elderly care sector of the healthcare organization to define their objective. They addressed the need for a dialogical approach to define what a good way of dealing with diversity could entail, discussed time and financial constrictions, and defined diversity as pertaining to sexual, cultural, and religious aspects of identity. Then the actual Socratic process began. The starting point was a SD that was facilitated for twelve team managers and their director during the first session by the first and last author. The clinical ethicists facilitated the SD according to the hourglass structure envisioned by Kessels (2001) and paid close attention to ensuring that participants had a Socratic attitude. All information provided by participants was denoted in bullet points on a flip chart during the dialogue. After an introduction round, the SD began.

Initially, all participants described concrete and positive personal examples of their own experiences with good ways of dealing with diversity in the elderly care sector. These examples where related to cultural and religious backgrounds, gender identity, and sexual orientation. However, they intersected with other diversity parameters. For instance, examples often also included considerations of age and generational differences (e.g., as these could influence perspectives on care or diversity), class (e.g., related to expectations and privileges) and ability (e.g., in terms of physical and cognitive (dis)abilities, and language skills). After sharing their examples, participants deliberated and eventually reached consensus about one case that they wanted to select for the joint reflection. This case was chosen because it was seen as a good example of dealing with diversity and associated social justice concerns in the elderly care sector. The case-presenter (CP) then described her case and elaborated on specific aspects in response to open and critical questions posed by the facilitators and participants. Her case was about a dinner that she had organized with healthcare professionals with sixteen different nationalities during which everyone brought food from their country of origin and they openly talked about their backgrounds and values. The conversation at dinner made her realize her own prejudices and stimulated a safer and more open work environment. Through joint dialogue, the participants then formulated a core statement—i.e., a response to the starting question—on what, in this example, was entailed in defining good ways to deal with diversity.

Participants subsequently deliberated on which rules they delineated from the example, while also recalling their own personal experiences with cultural, religious, and/or sexual diversity. They reached consensus on six essential rules for defining good ways of dealing with diversity in care for older people. The clinical ethicists supported this process by asking open and critical questions and reminding participants of their Socratic attitude. They also addressed participants that had been “dominant” or “quiet” for a while, to ensure that each voice was heard equally.

Subsequently, the participants reflected on which values and principles could be abstracted from these rules. This led to a consensual formulation of a preliminary definition—a response to the starting question of the SD—regarding what is entailed in good ways of dealing with diversity in care for older people. This initial definition was recorded on a whiteboard by one of the facilitators.

Box 1 Preliminary definition reached in session 1

**Table:** 

A good way of dealing with diversity in the care for older people starts with yourself. It is about continuously questioning yourself in relation to others. To do so, openness, curiosity, attention, and non-judgemental listening are important. Offering safety and trust for co-workers and patients is substantial to enable them to be themselves. It is not about thinking in stereotypes but about considering individual needs. To do so, patients have to be empowered and provided with freedom of choice and (care) offers and opportunities. Differences have to be accepted, knowledge and experiences shared, and by organizing shared activities, connection and equality can be achieved together. We are responsible, together, for dealing with diversity well.	

Note: summarized and translated from Dutch to English by first author.

The temporary definition was shown to five experienced diversity researchers between the first and second session, who provided feedback via email. The experts were recruited from the authors’ networks based on their expertise with various aspects of diversity. We use the term “expert” here to refer to the theoretical expertise of the researchers, which we see as complementary to the expertise of the dialogue-participants. The researchers specialized in gender diversity, cultural diversity, sexual diversity, participation, inclusion, interculturalization, and/or intersectionality.

These experts were included in the process to ensure that other relevant and critical voices were heard in the process of developing the diversity statement. They provided feedback on several aspects of the definition. They, for instance, stressed that more stakeholders from the work floor ought to be included in the process of developing the diversity statement and suggested that power dynamics, organizational responsibilities, and existing inequalities ought to be actively addressed within the organizational statement. The experts also argued that the definition focused too much on individual responsibilities of healthcare professionals and that the organization also had a key responsibility in facilitating diversity-responsiveness through recruitment and training.

During the second dialogical session, an experience-based, critical Socratic exploration and joint dialogue about the researchers’ feedback was carried out. In addition to the team managers, two client representatives and a policy advisor participated in this session. To promote open reflection and prevent the feedback from being perceived as authoritarian due to their expertise, the facilitators first presented the feedback by stressing that the researchers’ voices ought to be seen as equal to those of the participants present in the room. Then the participants reflected on the feedback amongst each other. They posed critical questions and provided arguments for their own perspectives on the issues the researchers addressed.

Several participants criticized the position of the experts that provided feedback. They, for instance, argued that because all experts were female, possessed a university degree, and were researchers, their expertise was limited and mostly academic. Eventually, consensus was reached about several additions to the conclusion of the first session (definition). For instance, the participants agreed that the expertise from “the work floor” was missing and necessary to develop a more inclusive diversity statement.

Between the second and third session, eight team managers consulted the experiential expertise of their team of different healthcare professionals (doctors, nurses, caregivers, etc. with various backgrounds) by facilitating dialogues on the temporary definition developed in the second session. Four team managers did not consult their teams because of a lack of time. The eight participants tried to engage in these group dialogues on the basis of Socratic epistemology that they had encountered in the first two sessions. The length of the dialogues differed, depending on practical time constraints in their specific settings. With participants acting as facilitators, we obtained insight on the relevance and applicability of the temporary definition from the perspective of the end-users— other healthcare professionals at the organization. They facilitated dialogues on the temporary definition to include the voices and perspectives of stakeholders from the work floor in the developmental process. They made notes of the critical feedback and questions addressed during the dialogical sessions.

At the beginning of the third session, i.e., the second Socratic exploration, the team managers who had engaged in a dialogue with their teams presented the main outcomes of their reflection processes, including the critical questions asked or suggestions offered by their respective healthcare team. Through an interactive dialogical process in which the similarities and differences between the dialogues and preferences of the teams were addressed, the participants reflected on the provided feedback. On the basis of this Socratic exploration, the participants eventually reached consensus on the final diversity statement. The diversity statement that was developed for the elderly care sector in this case example was later adopted as the general diversity policy of the long-term care organization. The diversity statement is summarized in box 1.

Box 2 Summary of the diversity statement

**Table:** 

A good way of addressing diversity in care occurs at the individual and at the organizational level. It encompasses knowledge development, care provision, and recruitment. A good way of dealing with diversity in care starts with yourself and with recognizing your own prejudices. It is about continuously examining these in relation to others by having an open, curious, and attentive attitude. Also team diversity is a strength, provided team members share knowledge and experiences and feel shared responsibility for establishing connection, safety, and equality. The organization is responsible for creating the (right) conditions for addressing diversity in a good way: it provides frameworks and support to employees and innovates within a constantly changing healthcare landscape. How organizational structures can create (in)equality or promote good ways of dealing with diversity needs to be scrutinized, addressed, and tackled. This is a joint task, which requires a top-down approach, vision, and responsibility, such as (openness for) bottom-up input, and for Othered voices. Regarding recruitment, the organization strives to ensure that societal diversity is represented at all levels. Also, [our organization] wants to actively promote cultural responsiveness, knowledge, and skills among employees. This involves organizing informal and formal activities. The guiding principle is that knowledge must be applicable, accessible, and transferred to a diverse group of employees. Regarding care services and care provision, a good approach to diversity means promoting patients’ freedom of choice by talking to patients and next of kin about care needs, rather than basing decisions on assumptions. Care options, services, and offers have to be actively presented to all patients in order to reach and inform them well. Diversity is neither a luxury nor a burden: diversity is our reality.	

Note: summarized and translated from Dutch to English by first author.

## Discussion

In this article, we explored how clinical ethicists may employ dialogical CES to support healthcare organizations with developing diversity statements. We argued that by taking a dialogical approach to CES through methods of MCD and the SD in particular, clinical ethicists can foster increased stakeholder participation and thereby promote equity and inclusion in the developmental process. We also provided a case example to illustrate what such a process may look like. Here we will discuss several benefits and challenges clinical ethicists may face when employing dialogical CES to support healthcare organizations with developing diversity statements.

### Benefits

Engaging clinical ethicists to develop diversity statements through dialogical CES has several benefits. As we have argued earlier, dialogue and ethical reflection can stimulate moral learning, foster critical consciousness about diversity issues and traditional healthcare practices, and promote diversity-responsive care (Abma et al. 2009; Abma, Leyerzapf and Landeweer 2016; Denier and Gastmans 2013; Erlen 1998; Frusti, Niesen, and Campion 2003; Kumagai and Lypson 2009; Metselaar et al. 2017; Pratt and DeVries 2018). Also, dialogical CES can increase stakeholder participation, especially given that the democratic imperative and dialectic interaction have been described as two central elements of participatory practice (Springett, Wright, and Roche 2011). Including minoritized and quiet voices in decision-making, policy development and research has been deemed important to stimulate diversity-responsive, inclusive, and equitable practices in healthcare and to facilitate outcomes that have practical relevance and benefit society (Abma et al. 2017a, b; Ghorashi and Sabelis 2013; Frusti, Niesen, and Campion 2003; Seeleman et al. 2015). This means that clinical ethicists can employ dialogical CES not only to increase participation and inclusion as an end in itself, but to support healthcare organizations to develop policies that reflect the needs of those the policies are about, which may increase their practical relevance and social impact.

In our case example, we show one possible way of how clinical ethicists can employ dialogical CES through an explorative Socratic process to develop a diversity statement in a way that includes more voices than traditionally present during a single MCD. In addition to the participants during the dialogical sessions, stakeholders from the work floor and external experts were consulted. While they did not partake in the dialogues themselves, their perspectives and feedback mattered and were reflected upon and incorporated into the final diversity statement. This meant that a variety of additional voices of otherwise potentially “forgotten” stakeholders were heard and included in the dialogical process, thus leading to the development of a diversity statement that represents a variety of relevant perspectives. This makes the Socratic process we described an example of how diversity statements can be developed dialogically and with additional stakeholder participation, other than only those partaking in a specific MCD. This can minimize epistemic injustice by fostering inclusion and improve the content and relatability of diversity statements.

In our case example, we also showed the role clinical ethicists can have in encouraging critical reflection and thereby transforming power relationships, empowering others, and reaching mutual understanding (see also Groot and Abma 2018) to generate a shared perspective on good diversity practices in healthcare organizations. As described by previous authors on participatory research processes: “the role of the ethicists is crucial, supporting reflection on the presuppositions of the interventions and dialogue about the results, either positive or negative. . . . the facilitator acts like a Socratic guide, questioning certainties and taken-for-granted assumptions” (Abma, Voskes, and Widdershoven 2017a, b, 150). By taking a hermeneutic-dialogical approach to CES as described in the case example, the clinical ethicists stimulated critical reflection on the Socratic process, personal judgements, and the “authority” of—for instance—the external experts throughout the SD and Socratic exploration sessions. The dialectic process encouraged participants to question presupposition and gave room to critical voices—particularly on aspects that can hinder the dialogical process or formulation of the diversity statement, like authority concerns.

Finally, including stakeholders that hold positions of power in dialogical CES can also benefit the development and implementation of organizational diversity statements. In our case example, the clinical ethicists collaborated with the director for the elderly care sector who also partook in the dialogical sessions. After the statement was developed, he presented it to the board of directors of the whole organization who eventually chose to adapt the statement as an organizational policy. Pratt and DeVries (2018) previously argued that health equity can be advanced by purposefully structuring deliberations among disadvantaged groups and those in power to change policy. By including the director, who had the power to address and facilitate organizational change, the outcome of the Socratic process found its way to being strategically implemented in organizational policy. The role of the clinical ethicist engaging in dialogical CES to develop diversity statements may thus also include reflecting on who needs to be included to achieve organizational change and implementation.

### Challenges

Despite the benefits of engaging in dialogical CES to develop diversity statements in healthcare, clinical ethicists also face several challenges. We would like to discuss these challenges in relation to our case example, by particularly focusing on concerns related to power dynamics and participation.

First, we must reflect on the role of the clinical ethicist when she is employed by a “client.” In our case example, the clinical ethicists that facilitated the dialogical sessions were recruited and paid by the organization. This client-service provider relationship raises questions regarding the ethicists’ independence and impartiality towards the content and dialogical process. Given as they were hired by the organization, they had an interest in facilitating CES in a way and achieving an outcome that was satisfactory to their employer. This interdependency may signal a power relationship that does not satisfy theoretical, dialogical—and participatory—ideals regarding the (financially) independent role of a clinical ethicist or facilitator in dialogical practice. This is a consideration that is immanent in instances where clinical ethicists facilitate CES with the goal of achieving a practical outcome (like a diversity statement) for a healthcare organization.

Second, another concern pertaining to power disparities in our case example, relates to engaging in CES deliberations among those in power (i.e., a director) and those that are dependent on those in power (i.e., a team manager or a client representative). Other scholars have argued that power differences can affect authenticity, equal participation, inclusion, and social impact and create epistemic injustice by excluding relevant but marginalized voices (Abma et al. 2001; Adler 2012; Boers 2005; Metselaar and Widdershoven 2016; Widdershoven 2001). In his discourse ethics, Habermas (1970, 1973) maintained that coercion- and power-free dialogue is essential to come to valid conclusions and to attain freedom and empowerment through dialogue with a group of interlocutors. Habermas argued that valid conclusions regarding moral obligations can only come about from a coercion-free dialogue (*herrschaftsfreie Kommunikation*) with all affected stakeholders. This can be achieved through what he describes as an “ideal speech” situation, where discourse occurs on the basis of consensus, inclusion, participation, and democracy (Habermas 1973). However, it may be difficult to apply ideals described in discourse ethics to dialogical practice and to eliminate authority, dependency, and power constraints completely. In our case example, the participation of the director during the Socratic sessions urges the question whether participants experienced complete freedom to be truly honest, open, and authentic about their thoughts and experiences (see also Boghossian 2002). It is possible that participants felt constraints that could have influenced their authenticity and honesty. The threat of authority or perception of power relations caused by the presence of their superior could have caused participations to seek approval and provide answers that they perceive as socially desirable (see also Grill et al. 2015). This may have tainted the dialogical process—a challenge that exists when engaging in dialogical CES that includes individuals with various backgrounds and positions within a hierarchical care organization.

Third, another consideration pertaining to power that is specific to our approach in the case example pertains to consulting an expert panel of researchers on preliminary results of the process. The term “expert” alone could have caused participants to view their feedback as valid, simply due to their perceived authority in the field of diversity. This threatens authenticity and equal contribution. To address this concern, the facilitators clarified during the dialogue that the “expert” voices were not voices of authority but as important as the individual voices present during the dialogue. Additionally, participants openly criticized the experts for having mere “academic expertise,” and argued that other expertise was also necessary for developing a diversity statement that is as inclusive of different voices as possible. This was why it was chosen to include other healthcare professionals in the process too. An important lesson here is that clinical ethicists or bioethics researchers engaging in dialogical CES on diversity issues have an important role to facilitate open communication, reflection, and criticism—especially about issues that may trigger authority-concerns (Abma, Voskes, and Widdershoven 2017a, b).

Rather than providing counterarguments to these reflections on power dynamics, we argue that, due to the inherently hierarchical structure of healthcare organizations and existing social inequities, power relationships will always exist to some extent: even in a dialogical setting. This creates the necessity to address these power issues with participants prior to, during, and after dialogical practice so that power imbalances can, to some degree, be counteracted. Previous scholars involved in participatory health research have made similar observations. Wilkinson et al. (2018) describe that while participatory research may be a more fair and equitable approach to research, we ought to remain “critical of the unresolved challenge of creating research equity. In particular (. . .) of power structures in participatory research . . . ” (1).

If we accept that the removal of power is a regulative ideal rather than being absolutely attainable, then a sufficient dialogical process is not about being able to guarantee the total absence of personal interests and ideal conditions but instead about dealing with and reflecting on them openly during the dialogue (also see Boers 2005; Metselaar and Widdershoven 2016; Metselaar and Widdershoven 2016). Recognizing and reflecting on the position of the clinical ethicists and all those involved in the dialogical CES process is crucial to being aware of positions of power, potential pitfalls, and bad-practices: an argument that has also driven participatory health researchers in striving to address power disparities in healthcare (Groot et al. 2019). This is also indirectly related to Mills’ assertion in “‘Ideal Theory’ as Ideology,” that moral theory—in general—is and ought always be located within the recognition that society and human interactions are shaped by power structures, social privileges, and disadvantages. This means that recognizing the role of systematic oppression and people’s social locations is a crucial first step in refraining from further perpetuating existing injustices and in changing social order. In his words: “one could say epigrammatically that the best way to bring about the ideal is by recognizing the nonideal, and that by assuming the ideal or the near-ideal, one is only guaranteeing the perpetuation of the nonideal” (Mills, 2005, 182).

Another challenge we would like to address relates to the issue of how inclusive and participatory dialogical CES can actually be. The key component of participation is including various stakeholders whilst paying special attention to marginalized voices in order to promote equity and increase the chance for social impact to occur (Abma et al. 2009; Abma, Leyerzapf and Landeweer 2016). In our case example, one may wonder to what degree the participation of marginalized voices was actually achieved. Most participants in the dialogical sessions had the same profession—they were team managers—also most were white women of Dutch descent. However, two client representatives and a policy advisor also participated, and we increased stakeholder participation—as opposed to traditional MCDs for instance—by consulting external experts and other care professionals from the work floor between the dialogical sessions as part of the Socratic process. Nonetheless, our participant make-up raises the question whether the quiet and most marginalized voices within the healthcare organization were sufficiently included. Rather, it is possible to argue that, despite our best efforts, the diversity statement was predominantly produced by those who have done well or fairly well within the bounds of the status quo and that some of the most marginalized remained marginalized from the process of generating the diversity statement, specifically from the three dialogical sessions. This is particularly concerning given our attempt to enhance social justice in the policymaking process and given that patient and community engagement, for example, is decisive in the development of diversity-responsive care and organizational practices—these are often the ones excluded and yet ultimately affected by a given policy or strategy (Seeleman 2015).

In response to this consideration, we would like to stress that this case example is a first exploration of how ethicists may be able to offer support in making healthcare settings more just in practice, by facilitating increased stakeholder participation in the development of a diversity statement through dialogical CES. This example, particularly regarding our participant make-up, is not ideal. Achieving “ideal” stakeholder participation in terms of including all minoritized voices in dialogical CES is a key challenge—and possibly unattainable theoretical ideal—for dialogical and participatory practices, especially in healthcare organizations where hierarchies, power disparities, and limited time and resources exist. However, clinical ethicists ought to continue to strive towards reaching participatory ideals on inclusion as much as possible, particularly when addressing diversity and justice issues. This entails recognizing and critically reflecting on which voices are and which voices are not represented, how this may be remedied, and what this may mean for (the outcome of the) dialogical process. Other clinical ethicists attempting to provide dialogical CES support to develop diversity statements in organizations should critically reflect on ways of expanding and selecting the group of those engaged in formulating a diversity statement, such that it more actively includes those in the organization pushed furthest to the margins, while being extra aware of epistemic injustices.

Furthermore, the main reason that team managers were included in this process was because of their strategic position, standing between healthcare professionals from the work floor and higher management. Their position allowed them to put diversity on the agenda within their teams (also after the statement was developed), to stimulate further dialogical reflection on the diversity statement in practice, and to act as “gatekeepers” that could transfer the voices from others in their teams to the dialogical table. However, we acknowledge that team managers not only hold a position of power but also benefit from material conditions that are not representative for the most marginalized people working for or receiving care in an organization. It is essential for clinical ethicists to reflect on and be aware of the considerations that come with limited and “not ideal” stakeholder participation. This concerns facilitating a deliberation process that aims to be as just and inclusive as possible, while fostering critical awareness on which voices are missing and on the effect this may have, and in developing a diversity statement that recognizes the need for continued dialogue and enhancing diversity-responsiveness and social justice above all.

Finally, we would like to end our discussion with a critical note from Ahmed’s work *On Being Included*. In her reflection on institutional diversity policies and documents, Ahmed describes that the way these documents are implemented in practice is more important than merely having “an amazing document” with great content that disappears in a drawer and is never seen or used again (Ahmed 2012, 6). She warns of the danger of diversity and equality becoming performance indicators or mere paper trails with little actual effect. Indeed having a diversity statement ought not become a substitute for social action and change. Nonetheless, engaging in dialogue and participatory practices with the intention of developing a diversity statement is a point of departure to foster necessary, recurring, and critical reflection about diversity issues, power, and social injustices. Further, critical dialogue on these topics ought to occur in different ways, with different stakeholders, and at all levels of an organization. However, a diversity statement, even when it is developed on the basis of participatory principles, is not in itself “good” or “sufficient” if it is not used, implemented, and practiced in a way that involves those it concerns and that continues to facilitate awareness, critical reflection, and ongoing dialogue on moral challenges related to diversity and social injustices.

## Conclusion

In this article, we argued that clinical ethicists can employ dialogical CES to support healthcare organizations in developing diversity statements in a participatory way. We explored what such a process may look like in practice. Taking a dialogical approach to developing diversity statements in healthcare organizations is important for facilitating more participation, diversity-responsiveness, and social justice and increasing the possibility for social impact. By engaging in dialogue with as many stakeholders as possible, more voices of those impacted by organizational policymaking can be included and epistemic injustice can be addressed.

We presented a case example in which a diversity statement pertaining to cultural, religious, and sexual diversity was developed for the elderly care context of a large long-term care organization by using a pragmatic, dialogical approach to CES. Specifically, we engaged in a dialogical process that consisted of one SD and two Socratic explorations with twelve team managers and their director, as well as client representatives. Between these sessions, additional stakeholders participated by providing feedback and reflecting on the preliminary outcomes of these dialogues.

From our explorations, we learn that it is challenging, and—from a theoretical perspective—possibly impossible, to attain ideals on power-free discourse, participation, and inclusion when developing diversity statements in practical settings. However, clinical ethicists may have a key role in striving toward these ideals: by facilitating a dialogical process that is as open, honest, and participatory as possible and in creating a space in which various (systemic) constraints such as hierarchy, scarce resources, power differences, organizational agendas, and inclusion and exclusion mechanisms are reflected upon. Ethicists ought to continuously reflect on the ambivalence of their role as clinical ethicists and service providers, their relationships with “clients,” and on their approach to stakeholder inclusion and participation, especially in a context where limitations in time and resources exist. Also they ought to facilitate reflection among participants about these issues.

We want to stress that the case example we describe is just one and not the ideal way of engaging in dialogical CES in healthcare organizations. Existing methodological plurality in CES methods also means that there is not one single good way for healthcare organizations to develop diversity statements. Further, this case example is an attempt to practically explore one way in which ethicists can facilitate more stakeholder participation in the development of a diversity statement. This case taught us that we need to critically reflect upon and further strengthen the participatory developmental process in the future, for example by including more marginalized voices during the dialogical sessions. Moreover, developing a diversity statement alone is not sufficient to address diversity and social justice issues well within healthcare organizations and does not necessarily mean that the statement is incorporated into practice or valued at different levels of an organization. Therefore, we suggest that addressing diversity well in healthcare organizations is a process of exploring moral ambiguity and differences in values and norms on diversity good practices. This means that dialogical practices on diversity ought to be an ongoing process in order to address moral challenges, stay alert to existing inequities, achieve and implement organizational change, and create a culture of dialogue. If healthcare organizations want to achieve and promote diversity-responsiveness at different levels, developing a diversity statement in a participatory way by engaging clinical ethicists to facilitate dialogical CES is only one step in what should be a continuous and structural attempt to promote inclusion and good diversity practices.
